# FDM 3D Printing of Polymers Containing Natural Fillers: A Review of their Mechanical Properties

**DOI:** 10.3390/polym11071094

**Published:** 2019-06-28

**Authors:** Valentina Mazzanti, Lorenzo Malagutti, Francesco Mollica

**Affiliations:** Department of Engineering, Università degli Studi di Ferrara, via Saragat 1, Ferrara 44122, Italy

**Keywords:** natural fibers, biocomposites, 3D printing, fused deposition modeling, mechanical properties

## Abstract

As biodegradable thermoplastics are more and more penetrating the market of filaments for fused deposition modeling (FDM) 3D printing, fillers in the form of natural fibers are convenient: They have the clear advantage of reducing cost, yet retaining the filament biodegradability characteristics. In plastics that are processed through standard techniques (e.g., extrusion or injection molding), natural fibers have a mild reinforcing function, improving stiffness and strength, it is thus interesting to evaluate whether the same holds true also in the case of FDM produced components. The results analyzed in this review show that the mechanical properties of the most common materials, i.e., acrylonitrile-butadiene-styrene (ABS) and PLA, do not benefit from biofillers, while other less widely used polymers, such as the polyolefins, are found to become more performant. Much research has been devoted to studying the effect of additive formulation and processing parameters on the mechanical properties of biofilled 3D printed specimens. The results look promising due to the relevant number of articles published in this field in the last few years. This notwithstanding, not all aspects have been explored and more could potentially be obtained through modifications of the usual FDM techniques and the devices that have been used so far.

## 1. Introduction

The 3D printing global market is growing at a very fast pace and is projected to expand even further in the next few years. Additive manufacturing techniques, in fact, are rapidly diffusing both in industrial and household settings, due to their many valuable characteristics [1]. If required production is limited to small scales, parts can be obtained easily [2,3,4], with limited scrap production and energy consumption [5], and without the need for expensive tools [6,7,8,9] or elaborate assembly [10]. Moreover, 3D printing techniques allow us to fabricate objects of complex shape or large thickness that are normally unobtainable through standard polymer manufacturing methods [11,12,13]. Finally, the inherent customizability is conveniently exploited in the biomedical materials field, with important applications in patient specific prosthetic devices or tissue engineering scaffolds [6,14,15].

**Figure 1:**
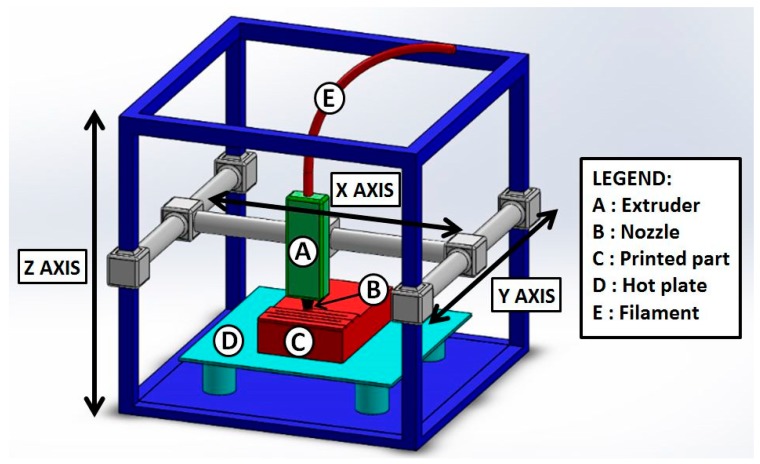
Schematic representation of a typical fused deposition modeling (FDM) setup.

Among the numerous 3D printing techniques, fused deposition modeling (FDM) is the most popular: It is conceptually simple, does not require health concerning solvents or glues [16] and most of all the printing apparatus is cheap and of small tabletop size [17]. The basic functioning of FDM is illustrated in Figure 1: A thermoplastic filament is continuously fed into a small heated chamber where it melts, becoming a highly viscous fluid, as it is typical of molten polymers, which are high molecular weight materials. The melt is then extruded through a nozzle and then deposed layer-wise on a heated table, following a pattern calculated by the printer control software that will reproduce the desired geometry of the object, which can be input as a CAD file, typically in STereo Lithography interface format (STL).

**Figure 2:**
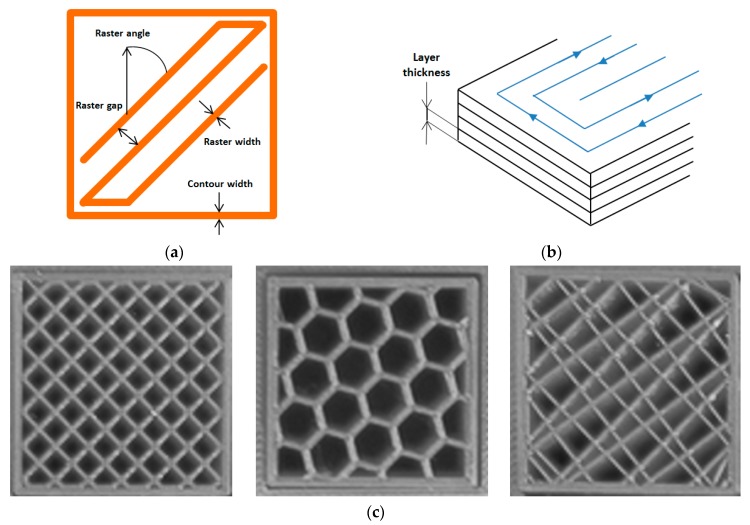
Structural parameters: (**a**) Parameters of the toolpath; (**b**) layer thickness; (**c**) infill geometry.

The FDM printing process depends on a large number of parameters, which are listed and briefly described in Table 1. For simplicity, these can be grouped into three macro-categories, namely (i) extruder-related, (ii) process-related and (iii) structural parameters. Filament width and nozzle diameter belong to the first group, while processing temperatures and printing speed are inside the second one. Perhaps, though, the most typical parameters are those belonging to the third category, which are also schematized graphically in Figure 2. Patterning is peculiar of FDM: It is the path followed by the nozzle to deposit the material onto the workspace. The most widely used toolpath is the so called “raster fill”: The layer contour is generated first, then the interior is filled following a back and forth pattern at a certain angle. After a layer is deposed, the subsequent one is filled after changing the raster direction.

From the materials point of view, thermoplastic polymers can be printed through FDM. Besides offering a clear environmental advantage in terms of recyclability, thermoplastics are the most common polymers and thus guarantee a greater choice of materials. These range from the so called “commodities”, like the polyolefins (i.e., polyethylene PE and polypropylene PP), passing through intermediately priced materials, such as acrylonitrile-butadiene-styrene (ABS), up to engineering plastics, which include polycarbonate (PC), polysulfone (PSU) or polyetherimide (PEI) and biodegradable materials like poly-(lactic acid) (PLA) [12,18]. Amorphous polymers are preferred over semicrystalline ones: Besides the lower solidification shrinkage, due to their liquid-like structure in the solid state, also their properties in the molten state are better. This can be explained in terms of melt viscosity that decreases with the distance between the processing temperature and the polymer glass transition temperature. In the case of semicrystalline thermoplastics, this distance is relatively high, as the processing temperature must exceed the crystal melting temperature, which is usually about 1.5 times the glass transition temperature expressed in °K. In amorphous materials, as there are no crystals, it suffices that the processing temperature be simply greater than the glass transition temperature by about a 100 °K.

Despite the many advantages of FDM, quite a few drawbacks are also intrinsically linked to this technology. For instance, the filament temperature plays a very important role in controlling the viscosity of the molten filament: This must not be too high, to allow easy flow through the nozzle orifice, yet it should not be too low, otherwise the deposed filament would not provide enough structural support for the subsequent layers [19]. Perhaps the biggest issue, though, is the mechanical properties of the final products [9,20,21]. These, in fact, are lower if compared with those of similarly shaped objects realized through standard processing methods (e.g., injection molding), due to the inevitable presence of voids [22]. Moreover, the mechanical properties of the printed object are anisotropic and highly dependent on processing parameters, as will be seen later [12,22,23,24].

Another important issue is the cost of FDM printed parts. This is influenced by the time to construct the product with the layer-by-layer building process, but also by the cost of the raw material [3]. In this respect, FDM is hampered by the relatively high monopolistic prices of the filaments, often protected by some restrictions placed by the printing machine producer [18]. It is thus highly desirable for a more widespread diffusion of FDM that filament prices be contained within reasonable bounds. Usually plastics cost can be reduced by using fillers, which are pulverized cheap materials, that can be added into the material formulation. Other useful improvements due to fillers are a certain increase in flexural stiffness and a better dimensional stability after solidification. On the other hand, such benefits come at the expense of a drastic reduction in tensile strength and an increase in density. Moreover, it is well known that wear induced by the relatively hard filler particles may severely limit the lifespan of plastics processing machineries, thus rising up their depreciation costs.

From this point of view, an interesting class of fillers is natural fibers, mainly of vegetable origin such as wood flour, rice or coconut husks, hemp or flax fibers and so on. These mainly consist of waste products coming from industry or agriculture, often produced locally [25,26]. Together with cost reduction, they clearly decrease the environmental impact of the compound, allow us to achieve lower density and are less stiff than traditional inorganic fillers, thus inducing negligible wear. Furthermore, they often impart a mild reinforcing effect, increasing the mechanical properties of the neat polymer. For this to hold, though, it is necessary to create a good interface between the polymer and the natural fiber, which can be achieved by performing a chemical treatment on the fiber surface [27,28] or also by compounding a suitable coupling agent in the polymeric material formulation [29,30,31].

For these reasons, natural fiber filled polymers, commonly known as “biocomposites”, are gaining important market shares in many relevant industrial sectors such as automotive, construction, thermal insulating and sound absorbing materials [32,33]. On the other hand, having natural fibers within the thermoplastic polymer poses a few peculiar problems. First of all, thermo-oxidative degradation of the biofiller, which is normally assumed to occur when the material is kept at temperatures above 200 °C for more than a few minutes [34]. This limits the choice of the polymeric matrix, as it must have a melting temperature that is rather low [35]. Moreover, the remarkable hydrophilicity of natural fibers requires that careful drying be performed prior to processing, in order to avoid water vapor development, or even worse hydrolysis of the polymeric matrices that are sensitive to this degradation phenomenon [36,37].

In FDM the use of thermoplastic biocomposite filaments is interesting for decreasing material cost [38,39] and environmental impact [3,38,40,41,42,43], reducing distortion after processing [44] while possibly preserving the mechanical properties of the material [45]. On the other hand, processing becomes difficult due to challenges shared with biocomposites processed with traditional methods, i.e., feedstock drying [12,46,47], inhomogeneity in filler dispersion, creation of voids during processing [19,48] and temperature control [49].

The problems described above indicate the possible reasons why such materials have appeared only in the most recent literature, i.e., mainly from year 2016 onwards. Nevertheless, the rate at which these articles are appearing denotes a remarkably growing interest from the scientific community and the availability of commercial biocomposite filaments testifies the interest of the market. The present review article aims at pointing out not only the many problems that are involved but also the most valuable achievements concerning the mechanical properties of these materials that will certainly play an important role in the future developments of FDM.

## 2. Methods

The selection of the articles analyzed in this review was made on the basis of certain specific characteristics. As Section 3 deals with the different materials that have been considered in the scientific literature, the papers that are studied had to be concerned with a thermoplastic polymer filled with natural fibers or particles, processed through FDM. In Section 4, the mechanical properties are presented, thus in addition to the previous requirements, the selected papers aimed at measuring tensile, flexural, compressive or impact properties of 3D printed samples. We found it convenient also to dedicate a subsection to papers that considered mechanical characterization of FDM filaments. Finally, Section 5 presents the discussion, conclusions and some possible future developments.

## 3. Materials

Natural fibers have been introduced recently as additives in FDM filaments [50]. In order to produce a good quality natural fiber filled thermoplastic filament, the biofiller must be well mixed within the polymeric matrix, like any other additive (e.g., coupling and toughening agents). This is obtained through compounding using a co-rotating twin screw extruder, which allows us to achieve both dispersive and distributive mixing [51]. The latter homogenizes additives uniformly within the matrix, while the former is important to eliminate additive clusters and is particularly relevant for natural fibers, as they tend to attract one another. Mechanical performance can also be improved by chemically treating the fibers, which has a positive effect on the load transfer capability of the biofiller-polymer interface [27].

The filament compositions and treatments that have been investigated in the scientific literature are summarized in Table 2, Table 3 and Table 4: Table 2 lists filaments based on non-biodegradable matrices, Table 3 is relative to biodegradable polymers, while Table 4 reports commercial filaments. For each material, the type, content and characteristics of the fibers, the possible presence of additives and the size of the filaments are detailed, together with the bibliographic references. As additional information, Table 2 and Table 3 report possible chemical treatments performed on the fibers and whether the processing parameters used to obtain the filaments are fully described. Concerning Table 4, the available information is only about matrix and biofiller type, declared loading percentage and the bibliographic references.

Despite a specific analysis of the filament materials is presented in the next two subsections, a general remark can be made on the fiber amount, which rarely exceeded 20–30 wt %. This is due to FDM printing becoming more complex: Melt viscosity increases with biofiller concentration, thus requiring higher power for extrusion through the nozzle. Moreover, sieving effects leading to nozzle blockage could occur [49] and this also poses bounds on filler size [23]. In addition, as the percentage of the polymer that can appropriately wet the fibers decreases, the filament may become too brittle [4]. Lastly, as the natural fiber quantity increases, the surface finish and consequently the dimensional tolerances are reduced [52].

### 3.1. Non-Biodegradable Polymeric Matrices

ABS is one of the most common materials in FDM filaments [53]: It is quite easy to print, exhibits adequate mechanical properties and toughness [7], high melt strength [4] and durability [19]. On the other hand, since ABS is not classifiable as a commodity, there is margin to decrease cost by adding cheap natural fibers [54]. In the last three years quite a good number of authors have investigated the effect of compounding bio-based fillers in ABS filaments [4,7,18,19,20,55]. From Table 2, it can be seen that ABS formulations are generally simple, with additives other than biofiller being only coupling [7,55] and toughening agents [4,7].

In order to decrease material cost even further, a limited group of researchers investigated polyolefins, such as PE and PP, which are true low cost commodities. In the pursuit of plastic waste reduction, pre-consumer PP waste was considered in [8,44], while for obtaining a more environmentally friendly material, bio-polyethylene (bioPE) was studied in [6,13]. This material is derived from vegetable feedstocks (e.g., sugar cane, sugar beet and wheat grain) and is chemically identical to petroleum based PE.

With polyolefins, the biggest issue comes from the printing process, as the mechanical properties of the melts are quite low. As described previously, this is typical of semicrystalline polymers and is due to the high difference between the polymer processing temperature, which obviously must exceed the crystal melting temperature, and their glass transition temperature. To limit these problems, in [8,44] a filament spooling machine was used to achieve good tolerance, as well as a 5 mm thick PP sheet retrofitted onto the print bed to improve adhesion. On the other hand, for these materials the effect of natural fibers becomes very interesting: Melt properties increase, thus improving self-sustaining characteristics during printing. Moreover, shrinkage and warpage effects decrease, but the poor adhesion between the polar fibers and the non-polar polyolefin matrix makes the addition of an appropriate coupling agent necessary (Table 2).

A last non-biodegradable biocomposite for FDM is thermoplastic polyurethane (TPU) [56]. TPU is interesting because of its versatility in terms of a wide range of mechanical properties, good abrasion resistance and low density [57]. However, cost is higher than other thermoplastics, therefore natural fibers can be helpful in making TPU more exploitable. Concerning additives, different types of coupling agents were analyzed in [56].

### 3.2. Biodegradable Polymeric Matrices

As the price of 3D printers drops every year, FDM becomes accessible to a growing number of people and this may increase the quantity of waste that is not properly disposed of or recycled at the end of life cycle. This is the main driving force for the development of filaments that are made of biodegradable or compostable materials [58].

PLA is the front-runner in the biodegradable plastics market, with the best availability and the most attractive cost vs. mechanical properties ratio [59]. Its production is relatively easy: It can be synthesized by condensation polymerization directly from lactic acid or from lactide ring opening, which are compounds that can be derived from fermentation of carbohydrate sources such as corn starch, sugarcane or tapioca [60]. Although it may possess a regular structure that would lead to a semicrystalline polymer, the most common commercial grades are almost completely amorphous glassy polymers.

The use of neat PLA in FDM 3D printing increases every year. It does not emit any unpleasant smell during the printing process and allows us to obtain components with reasonable tolerance [38]. The close connection between PLA and FDM has been established in the biomedical field, in particular in tissue engineering scaffold production. Here PLA can favor cell adhesion and proliferation, thus constituting a positive environment from a biological and mechanical point of view [61].

PLA biodegradation is due to hydrolysis: Water diffuses first into the amorphous domains and induces de-esterification. Degradation then proceeds also into the crystalline regions causing a drastic decay in the mechanical properties followed by complete dissolution of the material. On the other hand, as degradation is strongly accelerated by temperature, hydrolysis can be unwillingly activated during processing. Reaction kinetics is further accelerated by acidic environment, and as the degradation products lower pH, PLA hydrolysis is an autocatalytic process [36], thus it proceeds very quickly. PLA filled with natural fibers such as wood, hemp, kenaf and flax processed using standard technologies has already been studied [27,59,62,63]. Filler quantity and different fiber chemical treatments have a positive effect on stiffness and strength [27,28,64]. This however is closely linked to the compounding process, taking into account that natural fibers are highly hygroscopic. For these reasons, processing of natural fiber filled PLA is complex and requires appropriate feedstock drying and storage.

Although there are quite a few studies on PLA-based biocomposites in FDM, the challenges brought by the material coupled to the ones coming from the processing method have forced a “trial and error” procedure. Interestingly, the available literature that does not make use of commercial filaments is significant [3,9,17,21,23,38,39,45,46,48,49,65,66,67,68], but has appeared only recently and the effects of processing parameters, additives formulation, patterning and geometrical features are not yet deeply explored. As can be seen from Table 3, plasticizers [21,45,48], toughening agents [39,65] and compatibilizers [39] have been studied.

Besides PLA, other biodegradable polyesters were also considered in the literature, such as poly-(ε-caprolactone) (PCL) [16], poly-(hydroxyalkanoates) (PHA) [5] and their blends with PLA [12,20]. The growing interest towards biodegradable polymers filled with natural fibers is confirmed by the availability in the commercial filament market of biocomposite wood/PLA filaments, such as “Bamboo fill” and “Woodfill” (ColorFabb Company) and “Laywoo” (CC Products), which are listed in Table 4.

## 4. Mechanical Properties

Components produced through FDM have mechanical properties that are heavily dependent on printing architecture. For this reason, filament properties after compounding should be evaluated before introducing the numerous processing variables of 3D printing. Tensile, flexural, compressive and impact properties of 3D printed samples will be considered next and separately from each other, to allow for more significant comparison. In all published papers the specimens were shaped according to standards that are valid for materials in bulk. Despite this may seem inappropriate at first, one should consider that no specific standard for 3D printed parts exists.

### 4.1. Filaments

In the scientific literature a few researchers have dealt with the tensile characterization of filaments. Harakeke and hemp fiber filled PP was studied in [8,44] and was shown that both strength and stiffness increase with natural fiber content. Harakeke had a stronger effect than hemp, leading to a tensile strength increase of about 50% for a harakeke loading of about 30 wt %, while stiffness more than doubled.

The situation is quite different for PLA: 10 wt % wood flour filling led to a very small increase in strength [23], but it decreased remarkably at higher wood content. Interestingly, this could not be ascribed to PLA hygrothermal degradation, as the authors were careful in drying raw materials before processing. A similar situation was also found in cocoa shell waste (CSW) filled PCL [16] and in commercial wood/PLA filaments [69].

In order to explain this discrepancy, the chemical nature of the matrix may not be the only issue to consider. In fact, as reported in Table 2 and Table 3, the polyolefin filaments had a diameter greater than 2.4 mm, thus were significantly thicker than the polyester ones, whose diameter was 1.75 mm. Although this last size is the one that is most commonly required by 3D printers, it may not allow an adequate wetting of the fibers at high percentages of filler. Secondly, despite the similar fiber content (i.e., around 40 wt %) in both filaments, this is close to the maximum fiber loading for PLA, while polyolefins can accept wood filling up to 70 wt %. Notice also that the negative effect of biofiller is independent of filler geometry. In fact, Depuydt [48] et al. analyzed PLA filled with flax and bamboo fibers as a function of the length over diameter ratio (L/D) of the reinforcement. Despite their results showed that the L/D ratio had an important influence on stiffness (longer fibers increase it by 215%), it did not have the same influence on strength, and anyway the properties of neat PLA were much higher. The same authors evaluated the effects of two different plasticizers, finding no relevant influence and completed filament characterization with a verification of porosity reduction induced by vacuum drying at the end of compounding.

Concerning the effects of fiber treatment, Filgueira et al. [49] evaluated the strength of filaments based on PLA filled with thermomechanical pulp fibers (TMP), subjected to two different enzymatic modifications, namely laccase-assisted grafting (LG) and laccase-mediated grafting (OG). The OG-modified composites yielded the highest strength among all filaments, probably because of a better interfacial adhesion between TMP and PLA. On the other hand, strength of all other biocomposites was lower than that of neat PLA, and this was justified on the basis of filament porosity, which was confirmed by electron microscopy analysis.

### 4.2. 3D Printed Components

The mechanical properties of 3D printed parts depend also on a wide range of structural and printing parameters. These actually define a structure within the part, which is deeply connected with the material in making the properties of the printed component. These may change considerably even if only a single parameter is modified [70,71]. This is already known in the case of FDM of unfilled materials, as the mechanical properties are strongly influenced by the infill geometry of the specimen. The situation is even more complicated in the case of a composite, i.e., an intrinsically inhomogeneous and anisotropic material. This strong connection between structure, material and the final mechanical properties is still at the core of current research interests and needs further understanding.

The most frequently reported printing parameters are listed in Table 5. It can be seen that the most common nozzle diameter is 0.4 mm, albeit greater nozzles are also comprehensibly employed in the case of filled materials, up to 1.5 mm. The layer height is also centered around 0.2–0.3 mm, while the extrusion speed rate is more variable, ranging from 15 up to 100 mm/s. The extrusion temperature is always relatively high, i.e., greater than 180 °C, except for one paper, in which PCL was used, while the bed temperature is in the range 40–110 °C. For the reader’s convenience, also the commercial names of the 3D printers that have been used in the papers analyzed in this review are reported in Table 5, together with the bibliographic reference.

#### 4.2.1. Tensile Properties

**Figure 3:**
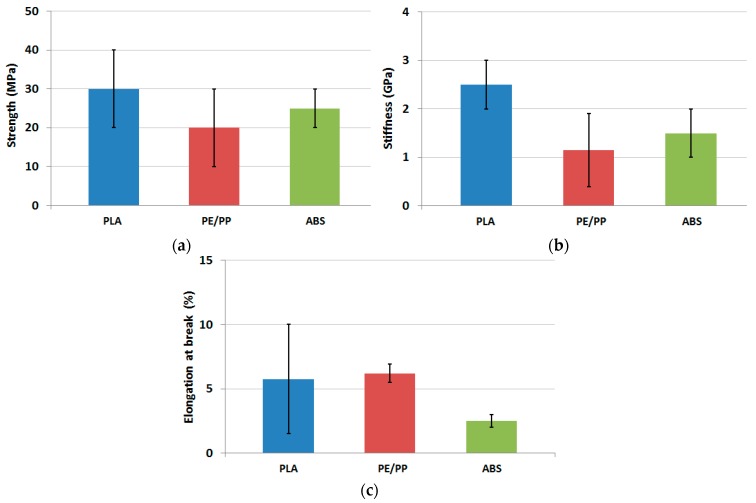
Average tensile properties of 3D printed samples for different polymeric matrices: (**a**) Strength; (**b**) stiffness and (**c**) elongation at break.

From Table 3 it is evident that the majority of the papers concerned with the tensile properties of FDM printed biocomposites dealt with PLA-based filaments [3,9,12,21,22,24,38,39,45,49,67,68,69,72,73,74]. These possess average tensile strength ranging from 20 up to 40 MPa, Young’s moduli between 2 and 3 GPa and elongation at break between 1.5% and 10%. The biocomposites based on ABS are the second class of materials [4,18,19,55]: Their properties range between 20 and 30 MPa for strength, 1 and 2 GPa for stiffness and 2%–3% for elongation at break. The third class is polyolefin-based biocomposites [8,13,44], which have strength around 20 MPa, Young’s moduli from 0.5 up to 2 GPa and elongation at break of about 5%. Strength, stiffness and elongation at break for these materials are reported in Figure 3. Only one paper investigated a TPU based composite [56], whose properties are similar to the other biocomposites, with the notable exception of elongation at break, which exceeds 300% at 40 wt % wood flour content.

**Figure 4:**
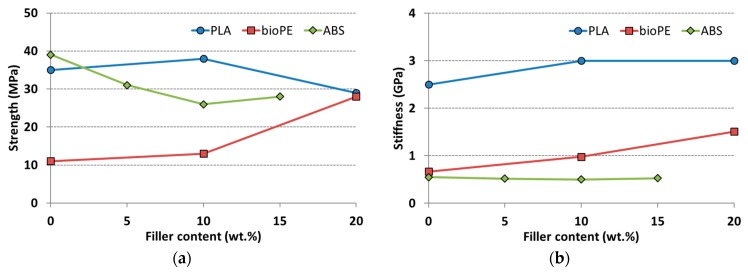
Mechanical properties of hemp/PLA [68], TMP/bioPE [13] and rice husk/ABS [19] as a function of biofiller content: (**a**) Strength and (**b**) stiffness.

Comparing unfilled printed specimens with reinforced ones, it can be often concluded that natural fibers have a negative influence on strength [3,4,9,18,19,38,39,56,67,68,69,73], while stiffness either increases slightly [8,13,18,19,44,68] or remains constant [3,38], as shown in Figure 4. The detrimental effect on strength is present in all materials, except for the polyolefins, where values increase with fiber content [8,13,44]. Analogously as filament properties, polyolefins do seem to benefit from natural fiber filling, irrespective of filler geometry: Indeed, TMP/PP biocomposite specimens increase their mechanical properties also with biofiller in the form of short chips, more similar to particles than to fibers [13].

The effect of additives other than biofillers has been studied only by a few authors [4,21,39,56]. Toughening agents, such as TPU on PLA/wood [39] or nitrile rubber on ABS [4], seem to improve strength, especially when coupled to compatibilizers. Notice, though, that strength of the composite remains lower anyway than that of the neat matrix. Xie et al. [21] evaluated the effect of two different plasticizers and their combination in different percentages and found that adding 4 wt % of tributyl citrate increased both strength and elongation at break. Bi et al. [56] tested different modifiers on wood/TPU composites, and found that it is possible to improve the interfacial adhesion between TPU and wood fibers with diphenylmethyl propane diisocyanate (MDI) and compensated the excessive flexibility with the addition of EPDM grafted with maleic anhydride (EPDM-g-MAH) as compatibilizer. Chemical treatments can also be used to enhance the properties of biocomposites [27,28]: Higher properties in sugarcane bagasse fiber (SBF) filled PLA are reported, in which SBF was treated sequentially with alkaline and acidic solutions [67].

Several authors have explored the effects that processing parameters have on the properties of the biocomposites [24,38,69,73,74]. On the basis of a design of experiment (DoE) methodology, Dong et al. [73] determined that the number of layers is the parameter that mostly affects the tensile properties, and verified this finding in the case of unfilled PLA and wood/PLA systems. The effect of the printing layer thickness was studied in [74] for a commercial wood/PLA, yielding the best results with a 0.05 mm layer thickness (i.e., the minimum tested value) and this was explained due to reduced porosity. Again for the wood/PLA system the negative effects of high processing temperature were investigated in [24]: At temperatures higher than 200 °C, strength decreased by 10%, while stiffness dropped by 5%. The infill density is also a very important parameter, as reported in [69], for pure PLA and two commercial wood/PLA filaments. As expected, infill density increased the mechanical properties for all materials, but more effectively for pure PLA than for the composites. Instead, nozzle diameter (0.2 mm up to 0.4 mm) was studied in [38], with 0.4 mm providing maximum elongation at break and strength but lower stiffness. Sample width can have a strong effect on tensile properties [12], and this is related to the percentage of overlap, and hence porosity, of the samples. Patterning has a significant effect [12,19,67], in that specimens printed following a longitudinal toolpath are much stiffer and stronger than the ones printed following other directions [12], due to poor interfilament adhesion [67]. Interestingly, though, increasing the filler content had a particularly negative effect for the 45° raster angle specimens, while the Young’s modulus for the 0° angle remained almost constant at any percentage of filler [19]. Environmental conditions can also modify the behavior of wood/PLA FDM printed samples [12]. In hygroscopic saturated conditions stiffness and strength were reduced by about 12.5% and 25%, respectively.

In an interesting group of papers, the tensile properties of 3D printed biocomposite specimens were compared to those of samples obtained by processing the same materials through injection molding [13,18] and compression molding [38,45]. Stiffness and strength of 3D printed specimens were similar to compression molded ones, but much less than injection molded samples. This may be clearly justified on the basis of the lower forming pressure of compression molding with respect to injection molding, but this conclusion cannot be considered completely exhaustive, as too few results are present in the literature, thus further investigations are necessary.

Hinchcliffe et al. [72] and Matsuzaki et al. [22] investigated continuous natural fiber reinforcement on 3D printed specimens in the form of an I-beam and a plate, respectively. The stress-strain behavior studied in [72] demonstrated that prestressed flax fibers strands increased the ultimate strength and stiffness of the samples, while jute fibers did not induce a significant improvement over the unreinforced specimens. Instead, in [22] the modulus and strength of jute reinforced plate specimens were +157% and +134%, respectively, compared with PLA specimens, but the 3D printer that was used did not allow fiber pre-tensioning.

#### 4.2.2. Compressive Properties

Compressive properties of FDM printed natural fibers reinforced thermoplastics were studied only in three articles, i.e., [24,55,75]. The range of average values of strength goes from 15 MPa for ABS based composites up to 30 MPa for those based on PLA. Compressive failure is primarily determined by localized buckling of the outside layers, as reported for all materials studied in [55]. The same type of failure was observed in [24], where the effect of printing temperature on strength was evaluated for a commercial wood/PLA filament. For compressive properties the effect of a temperature increase seems to be beneficial, as there is an improvement by 15% in strength in going from 200 °C up to 230 °C. Tao et al. [75] evaluated the influence of infill geometry using a commercial wood/PLA filament. This study compared the performance of different 3D printed cellular structures depending on the geometry of the cell cavities (circular, square and voronoi), indicating that square geometry is the most rigid, but the authors pointed out that square paths are inherently easier to print successfully. The influence of the deposed line width was also evaluated, with greater line width creating higher porosity, thus lower properties.

#### 4.2.3. Flexural Properties

Almost all of the literature that studied the flexural properties of biocomposites in FDM used PLA as the matrix [24,39,46,66,67,72,73,74] with only one exception, i.e., [19], in which ABS was used. Flexural properties were always measured through the three-point bending method and ranged between 30 and 60 MPa for strength and between 2 and 4 GPa for stiffness.

In analogy with tensile properties, flexural strength rapidly decreases with the filler content [19,39,67], while the situation is different for stiffness. Here, the literature explored only the range from 0 wt % up to 15 wt % filler content, finding that the Young’s modulus increases by about 20% or more [19,67].

The effect of additives was studied only in [39], where different toughening agents were evaluated (Table 3). In keeping with tensile properties, the addition of TPU seemed to be the best choice to increase strength. The authors also studied the effects of two different coupling agents, but these failed to improve strength. Fiber chemical treatment is beneficial also in the case of flexural properties [67].

FDM process parameters were investigated only in three papers [24,73,74]. The number of layers was found to be more effective than infill density and layer thickness [73]. Interestingly, though, unlike tensile properties, this effect was found to be more significant in the case of composites rather than the neat matrix. Ayrilmis et al. [74] investigated the effect of printing layer thickness on the bending properties of a commercial wood/PLA filament, finding significant improvement at minimal thicknesses because of reduced porosity. The effect of extrusion temperature on wood/PLA composite was studied in [24]: This parameter can be critical during the printing phase because both natural fibers and PLA can suffer from thermal degradation. Stiffness appeared to decrease with printing temperature, while strength seemed to remain about constant.

In addition to material and processing variables, also environmental parameters can influence flexural properties. Kariz et al. [46] evaluated the behavior of wood/PLA samples in different humidity conditions (i.e., 33%, 65% and 87%), finding that stiffness of the wood filled composites strongly decreased with moisture content.

Lastly, concerning continuous reinforcement, natural fibers inserted within a FDM printed I-beam [72] or a plate [66] are found to increase the flexural properties of the samples, but the effect depended on the prestress level superimposed to the fibers prior to insertion into the FDM printed part.

#### 4.2.4. Impact Properties

Adding natural fibers to an unfilled thermoplastic polymer processed with standard technologies normally reduces its impact properties [76], since filler introduces defects and stress intensity regions that may embrittle the material, even if chemical bonds between fibers and matrix are adequate. In the published literature concerning impact properties of FDM, only wood/PLA biocomposites have been evaluated. Accordingly, it was found that toughness always decreased when adding natural fibers or particles [69] and this was particularly significant when compared with unfilled PLA [39,65,73].

The effect of toughening agents was studied in [39,65]. In particular, Guo et al. [39] concluded that TPU is more effective than PCL and metallocene-based poly(ethylene-octene) elastomer (POE). POE was studied also in [65] and was found to improve impact properties in such a way that a 15 wt % content was sufficient to recover the impact strength of neat PLA. Coupling agents are studied only in [39], where it is reported that the combined effect of an appropriate compatibilizer with a toughening agent is effective.

Only two papers have studied the influence of processing parameters on impact properties. Increasing the number of layers had no effects on wood/PLA biocomposites, in spite of being advantageous in neat PLA [73], while the infill density (from 23% up to 55%) had a positive effect on commercial wood/PLA [69].

## 5. Discussion and Conclusions

FDM 3D printing is still a relatively new manufacturing technique and much research aims at improving printed products by investigating processing and structural parameters, but also materials in terms of additives. Natural fiber filling could be beneficial to decrease filament cost, yet retaining mechanical properties. Moreover, when a biodegradable polymer is used as the composite matrix, natural fibers do not alter biodegradability in the final material.

The scientific research that has been conducted so far has tackled many problems connected with the use of biocomposite filaments, but successful solution of relevant issues requires a well-established expertise in both fields, i.e., FDM technology and natural fiber filled polymers. Nevertheless, a remarkable number of excellent research articles have been produced in the last 2–3 years, therefore it is appropriate to take stock of the current situation.

Concerning the mechanical properties of 3D printed materials, there is no recognized international standard that regulates the characterization of their tensile, compressive or flexural properties. Papers dealing with the quantification of mechanical properties followed specimen dimensions and size that reproduce those that are used for the characterization of polymeric materials in bulk (e.g., dogbone specimens). In this case, geometric characteristics are normalized out through the concepts of stress and strain, but in the case of 3D printing this is difficult because the specimen is actually a structure (Figure 2), not a material. For instance, even the simple identification of the cross sectional area is non-trivial. For such reasons, the use of geometries and methods specified for materials in bulk is not fully justified in the case of 3D printed materials. A further complication comes from the influence of processing parameters (Table 5) that also play a role in the determination of the mechanical properties of 3D printed materials, thus making it difficult to draw conclusions that can be considered sufficiently general, in particular when comparing results from different papers.

This notwithstanding, it appears that, at least for ABS-based and PLA-based materials, the addition of natural fibers has a negative effect on the mechanical properties: Strength always decreases with filler content, while stiffness remains basically equal to that of unfilled material at low amount of biofiller, but it decreases at higher loadings. Elongation at break also decreases, thus determining a general embrittlement of the biofilled material. Interestingly, though, materials that are not extremely common in FDM (i.e., PE and PP, because of their semicrystalline nature) do receive benefit from compounding with natural fibers or particles. This aspect definitely should not be underestimated, thus it would certainly be interesting to investigate further with other semicrystalline polymers.

Filler geometrical characteristics are known to play an important role in the mechanical properties of the resulting composites. This is true also for natural fiber filled polymers [77,78]. In the FDM specific literature, fiber morphology information is often incomplete, as it is usually constituted by average particle dimensions, often coming from specification of the mesh size before compounding. On the other hand, more precise quantities are more relevant for determining the performance of fiber reinforced materials: The fiber aspect ratio L/D is one of the most important geometrical parameter, yet a specific study in FDM of natural fiber filled polymers is present only for filament tensile testing [48].

Toughening agents were found to be beneficial for impact properties but also for tensile and flexural strength. This can be explained because this additive creates a multiphase structure, reducing stress concentration and absorbing a large quantity of energy at impact. Moreover, as toughening agents have low viscosity, they can help in filling voids, reducing porosity and also improving the fiber-matrix interfacial bonding.

There is also wide agreement that important difficulties arise when printing filled materials. These include, but are not limited to, filler induced viscosity increase, matrix-filler wetting and fiber-matrix interface issues. Extrusion through a relatively narrow nozzle may also give rise to sieving phenomena, especially in the case of a large sized filler. This can potentially be troublesome, as it would cause irregular material flow through the extruder and this may in turn induce defects in the 3D printed part.

If natural fibers are used as fillers, processing can be worsened by specific issues. Firstly, one should consider careful material drying, definitely prior to the compounding phase, but possibly also before printing. This is important to reduce the amount of water vapor that is brought into the material by the hydrophilic natural fibers, a pitfall that is essential to avoid in the case of thermoplastics that are sensitive to hydrolytic degradation. Secondly, natural fiber thermo-oxidative degradation must be prevented by processing biofilled materials at temperatures that are not in excess of 200 °C. This issue is particularly complex in the case of highly filled materials: As remarked previously, material viscosity may increase considerably, thus making it necessary to set high values of the extrusion temperatures (see Table 5). On the other hand, even if the extruder temperature is set above 200 °C, the reduced permanence time of the melt inside the heated chamber may prevent biofiller degradation due to the low thermal conductivity of molten polymers. Hence, degradation phenomena can be controlled also through the printing speed that must be sufficiently fast that material permanence at dangerous temperatures is minimized. Another important point to consider could be the usage of external lubricants, which is extremely common in natural fiber filled polymers processed through standard methods, although it may give rise to particular wall slip phenomena [79]. From this point of view, research towards material formulations that modify heat transfer or flow properties would be highly desirable and should be considered as future developments of this field. Needless to say, processing difficulties may have important negative consequences on the mechanical properties of the final product.

In any case, an important point is to check carefully the quality of the filament prior to printing. This must not contain voids or other defects and its composition should also be verified in terms of additives and natural fibers concerning their quantity, morphology and distribution. Suitable methods include, for example, scanning electron microscopy imaging and dissolution of the biocomposite matrix in a solvent followed by filtration to isolate natural fillers. The same procedure could also be performed on printed components.

Many drawbacks could be controlled by carefully choosing the processing parameters, but it must be pointed out that FDM, even in the case of unfilled materials, is characterized by a large number of variables, thus it is difficult to pinpoint the ones that mostly affect structure/properties correlations. Moreover, parameters influence may also be material dependent and interconnected among each other, thus further investigations in this direction for natural fiber filled 3D printed polymers are definitely necessary.

**Figure 5:**
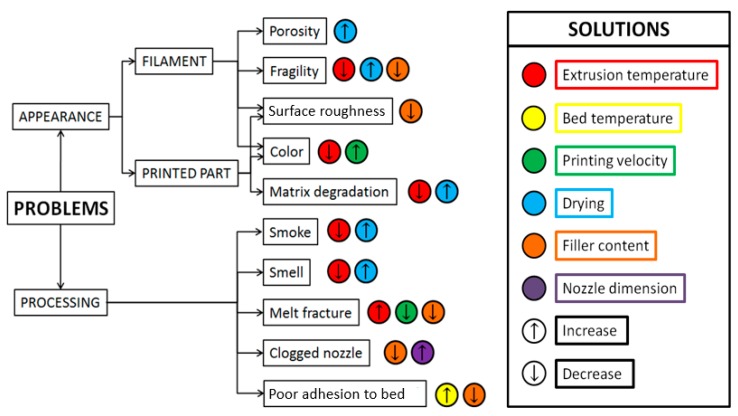
Natural fiber specific problems during FDM and their possible solutions.

Nevertheless, for the reader’s convenience, a troubleshooting guide is pictured in Figure 5. Concerning the problems presented therein we concentrated only on natural fiber related ones, and refer to other articles, e.g., [80], for more general issues arising in FDM 3D printing. A first classification is presented based on problem localization, and a list of possible solution is proposed. Among these, the easiest to be implemented are those referring to processing parameters, such as printing speed, extruder and bed temperatures, but also machine- and material-dependent parameters are considered, such as the nozzle diameter and the filler content.

Interestingly, in the scientific literature published so far the printing apparatus is usually a standard off-the-shelf device, not optimized for processing biofilled materials. It is likely that better quality biocomposite printing might be obtained with specifically designed printing machines.

## Figures and Tables

**Table 1:** Description of the printing parameters.

Parameters		Description
Extruder Geometry	Nozzle diameter	Size of the exit orifice of the extruder
	Filament diameter	Size of the filament required by the extruder
Processing	Melt temperature	Temperature of the molten material exiting the extruder
	Hot plate temperature	Surface temperature of the workspace plate
	Printing speed	The velocity of the material deposition
Structural	Layer thickness	The thickness of the layer deposited by the nozzle
	Infill geometry	The internal structure of the printed component
	Infill density	Material percentage filling the component apparent volume
	Number of layers	Number of shells deposed
	Raster angle	The angle between the deposed material and the x-axis
	Raster gap	The distance between two contiguous paths on the same layer
	Raster width	Width of the deposed material
	Patterning	Path followed to deposit the material on the workspace

**Table 2:** Non-Biodegradable filaments.

Matrix	Matrix Trade Name	Filler	Filler Trade Name	Filler Content (%)	Filler Chemical Treatment	Compatibilizer	Toughening Agent	Diameter (mm)	Test Type ^1^	Ref.
ABS	Scientific Sales, Inc.	hardwood lignin + carbon fibers	Lignol Innovations	20–40 + (CF10)	/	/	Nitrile rubber	2.5	T	[4]
	Magnum 3616 7 NAT HTP	coir fibers	/	15	/	/	/	1.75	T	[18]
	Taitalac 5000s	rice straw	Local farmlands	0–15	/	/	/	1.75	T-F	[19]
	Not Specified	macadamia nutshells	/	19–29	/	MAH 3 wt %	/	1.75	T-C	[55]
PP	Astron Plastics	hemp	Hemp Farm NZ Ltd.	10–30	Alkaline	MAHg-PP 2 wt %	/	2.4–3.1	FT-T	[8]
	Astron Plastics	harakake	Templeton Flax Mill	10–30	Alkaline	MAHg-PP 2 wt %	/	2.4–3.1	FT-T	[8]
	Astron Plastics	hemp	Hemp Farm NZ Ltd.	0–30	Alkaline	MAHg-PP 2 wt %	/	3	FT-T	[44]
	Astron Plastics	harakake	Templeton Flax Mill	0–30	Alkaline	MAHg-PP 2 wt %	/	3	FT-T	[44]
	Astron Plastics	gypsum	Gib Plasterboards	0–30	Alkaline	MAHg-PP 2 wt %	/	3	FT-T	[44]
bioPE	Braskem	TMP	Norske Skog Saugbrugs	10–20	Lauryl Gallate Octyl Gallate	MAH-PE	/	2	/	[6]
	Braskem	TMP	Norske Skog Saugbrugs	10–20	BioPE solubilisation	MAH-PE	/	/	T	[13]
TPU	Deansheng Plastic Company	poplar wood flour	Lingshou County Mineral Plant	10–40	/	EPDM-g-MAH, POE-g-MAH, chitosan, MDI 5 wt %	/	1.75	T	[56]

^1^ FT: Filament Testing, T: Tensile, C: Compressive, F: Flexural, I: Impact.

**Table 3:** Biodegradable filaments.

Matrix	Matrix Trade Name	Filler	Filler Trade Name	Filler Content (%)	Filler Chemical Treatment	Compatibilizer	Toughneing Agent	Plasticizer	Diameter (mm)	Type of Test ^1^	Ref.
PLA	/	Paulownia wood	/	25	/	/	/	/	1.75	T	[3]
	/	Orange wood	/	25	/	/	/	/	1.75	T	[3]
	Ingeo 4032D	Aspen sawdust	Laboratory	5	/	/	/	/	1.75	T	[9]
	Ingeo 4032D	Bamboo	Faber-Castell	20	/	/	/	PEG600 Ester	1.75	/	[17]
	/	Poplar wood	/	30	/	/	/	Glycerol Tributyl citrate	1.75	T	[21]
	Ingeo 2003D	Wood powder	Laboratory	0–50	/	/	/	/	1.75	FT-F	[23,46]
	Ingeo 2003D	Pine lignin	MWV Chemicals	5	/	/	/	/	1.75	T	[38]
	Ingeo 4032D	Poplar wood	/	10	/	Graft copolymers Glycidyl methacrylate Dicumyl peroxide	TPU, POE 10 wt %	Aliphatic polyesters 10 wt %	/	T-F-I	[39]
	Ingeo 4032D	Cork powder	Amorim Revestimentos	5	/	/	/	Tributyl citrate 5 wt %	1.75	T	[45]
	/	Bamboo	Bambooder Fibers	15	/	/	/	cPLA1–cPLA2	2.85	FT	[48]
	/	Flax	Lineo	15	/	/	/	cPLA1–cPLA2	2.85	FT	[48]
	Ingeo 4043D	TMP	Norske Skog Saugbrugs	10–20	Lauryl Gallate Octyl Gallate	/	/	/	2.2	FT-T	[49]
	Ingeo 4032D	Poplar wood	/	0–10	/	/	POE	/	1.75	I	[65]
	Ingeo 4032D	Sugarcane	Guangzhou Inst.	3–15	Alkaline	/	/	/	1.75	T-F	[67]
	Ingeo 3052D	Harekeke	Templeton mill	0–30	Alkaline	/	/	/	/	T	[68]
	Ingeo 3052D	Hemp	Hemp Farm	0–30	Alkaline	/	/	/	/	T	[68]
PHB	Biomer	Sawmill	Local	20	Enzymatic saccharification	/	/	/	1.75	/	[5]
PCL	Polysciences	Cocoa shell	Ferrero S.p.A.	0–50	/	/	/	/	1.75	FT	[16]
PLA + PHA	/	Cellulose pulp	/	/	/	/	/	/	1.75–3	/	[41]

^1^ FT: Filament Testing, T: Tensile, C: Compressive, F: Flexural, I: Impact.

**Table 4:** Commercial filaments.

Matrix	Filler	Filler Content (%)	Commercial Name	Diameter (mm)	Type of Test ^1^	Ref.
PLA	/	/	Verbatim	2.85	/	[20]
	Cedar fibers	40	EasyWood	1.75	T-C-F	[24]
	Cellulose	40	Laywood	/	/	[47]
	Wood	30	Not specified	1.75	/	[52]
	/	0	PLA PrintPlus	1.75	FT-T-I	[69]
	Recycled wood	30	WoodFill fine	1.75	FT-T-I	[69]
	Recycled wood	40	Laywoo-D3	1.75	T-I	[69]
	Wood	40	Bilby 3D	1.75	T-F-I	[73]
	Wood	30	Not specified	1.75	T-F	[74]
	Wood	/	Not specified	1.75	C	[75]
PLA + PHA	Recycled wood	15	ColorFabb	2.85	T	[12]
	Bamboo	20	ColorFabb	2.85	/	[20]
ABS	/	/	Verbatim	2.85	/	[20]

^1^ FT: Filament Testing, T: Tensile, C: Compressive, F: Flexural, I: Impact.

**Table 5:** 3D printing FDM parameters.

Nozzle Diameter (mm)	Extrusion Temperature (°C)	Bed Temperature (°C)	Extrusion Speed Rate (mm/s)	Layer Height (mm)	3D-printer	Ref.
0.4	220	70	90	0.34	MakerBot-Replicator 2	[3]
0.5	230	110	50	/	LulzBot TAZ	[4]
0.75	190	40	25	0.3	MakerGear™ V2	[5]
0.4	210	/	15	/	Ultimaker Original	[6]
/	210	/	/	/	da Vinci 1.0	[7]
1.5	230	/	/	1	Diamond Age	[8]
0.4	210	/	/	/	Self-assembled	[9]
0.4	210	70	18	/	Prusa i3-Rework	[12]
/	180–200	/	/	/	Prusa i3	[13]
0.6	120	/	50	0.3	Prusa i3-Hephestos	[16]
/	190–195	/	30–50	/	/	[17]
/	230-245	70	21	0.2	Easy3DMaker	[18]
1	250	100	/	0.2	Printrbot Simple Metal	[19]
/	220–275	60–90	30–40	0.4	CreatBot DX-3D	[20]
/	220	/	/	/	MakerBot-Replicator 2	[21]
/	210	80	60–100	/	Blade 1	[22]
0.4	230–275	/	30	0.19	Zortrax M200	[23]
0.4	200–230	50	30	/	Creator Pro-Flashforge	[24]
/	205	/	20	0.1	Zmorph 2.0	[38]
/	/	/	/	/	MR300	[39]
1	230	/	50	/	/	[44]
0.8	230	60	30	0.4	MakerBot-Replicator 2	[45]
0.4	230–275	/	30	0.19	Zortrax M200	[46]
/	/	/	/	0.8	/	[47]
0.4	210	/	15	/	Ultimaker Original	[49]
0.4	200	80	/	0.05–0.3	Zaxe	[52]
0.5	/	/	/	/	Leapfrog Creatr	[55]
/	185	/	/	/	MR300	[56]
0.5	180	/	/	0.1	/	[65]
0.4	/	/	/	/	Accucraft	[66]
0.6	200	50	40	0.1	/	[67]
1	/	110	/	/	Diamond age	[68]
0.5	188	50	60	0.4	Profi3Dmaker	[69]
/	215	/	60	0.2	MakerBot-Replicator 5	[72]
/	230	70	90	/	MakerBot-Replicator 2	[73]
0.4	200	80	/	/	Zaxe	[74]
0.4	200	60	30	0.3	Open source, 605 S model	[75]
